# “The Penny Drops”: Investigating Insight Through the Medium of Cryptic Crosswords

**DOI:** 10.3389/fpsyg.2018.00904

**Published:** 2018-07-03

**Authors:** Kathryn J. Friedlander, Philip A. Fine

**Affiliations:** Department of Psychology, University of Buckingham, Buckingham, United Kingdom

**Keywords:** cryptic crossword expertise, Aha! insight problem-solving, representational change, chunk decomposition, opportunistic assimilation, rebus and remote association puzzles, jokes, anagrams

## Abstract

A new protocol for eliciting insight (“Aha!”/Eureka) moments is proposed, involving the solving of British-style cryptic crosswords. The mechanics of cryptic crossword clues are briefly explained, and the process is set into the insight literature, with parallels being drawn between several different types of cryptic crossword clues and other insight-triggering problems such as magic, jokes, anagrams, rebus, and remote association puzzles (RAT), as well as “classic” thematic or spatial challenges. We have evidence from a previous survey of cryptic crossword solvers that the “Aha!” moment is the most important driver of continued participation in this hobby, suggesting that the positive emotional “payback” has an energizing effect on a participant's motivation to continue solving. Given the success with which a good quality cryptic crossword elicits “Aha!” moments, cryptics should prove highly valuable in exploring insight under lab conditions. We argue that the crossword paradigm overcomes many of the issues which beset other insight problems: for example, solution rates of cryptic crossword clues are high; new material can easily be commissioned, leading to a limitless pool of test items; and each puzzle contains clues resembling a wide variety of insight problem types, permitting a comparison of heterogeneous solving mechanisms within the same medium. Uniquely among insight problems, considerations of expertise also come into play, allowing us to explore how crossword solving experts handle the deliberate misdirection of the cryptic clue more effectively than non-expert, but equally experienced, peers. Many have debated whether there is such a thing as an “insight problem” *per se*: typically, problems can be solved with or without insight, depending on the context. We argue that the same is true for cryptic crosswords, and that the key to the successful triggering of insight may lie in both the difficulty of the challenge and the degree to which misdirection has been used. Future research is outlined which explores the specific mechanisms of clue difficulty. This opens the way to an exploration of potential links between solving constraints and the experiencing of the “Aha!” moment, which may shed light on the cognitive processes involved in insight solution.

## Introduction: insight and “insight problems”

The feeling of insight—a sudden, euphoric “cognitive snap” (Weisberg, 2015) signaling a breakthrough in the solution of a problem—is well-known to most of us. In terms of its phenomenological experience, four key elements of the insight, or “Aha!” moment have been identified: first, the suddenness and unexpectedness of the resolution, which arrives unheralded by conscious awareness of the solution path or “feelings of warmth” at the approaching dénouement; secondly that—however difficult it had proved before (perhaps involving a state of impasse)—the problem can be rapidly processed once the solution has been identified; thirdly that there is a strong, typically positive, emotional response at the point of resolution; and finally that the solver is fully convinced that the correct solution has been identified (Topolinski and Reber, 2010a; see also Metcalfe, 1986; Davidson, 1995; Gick and Lockhart, 1995; Danek et al., 2014a,b; Kounios and Beeman, 2014; Shen et al., 2015; on negative insight (“Uh-oh”) see also Hill and Kemp, 2016). The phenomenological experience of the “Aha!” moment is thus complex, with at least four contributory components: suddenness, surprise, happiness and certainty (Gick and Lockhart, 1995; Danek et al., 2014a, 2016).

One of the key problems in studying insight is the unpredictability of this moment in everyday life. Although “everyday insight moments” can be experienced (such as the sudden realization of where a bunch of keys has been left), the sudden and fleeting nature of this moment has led most studies to attempt to elicit responses artificially under laboratory conditions, using a bank of so-called “insight problems” intended to trigger the identical phenomenological response (Hill and Kemp, 2016). Nonetheless, even this approach is not without issues, primarily centered upon the difficulty of finding an effective, convenient, and reliable insight-triggering task for the participant to solve.

### Current obstacles in exploring insight in the laboratory

Lab studies of insight in problem solving have met with a number of obstacles, which have been well rehearsed in the literature. These include the historic paucity of standardized problem material (MacGregor and Cunningham, 2008; Batchelder and Alexander, 2012; Danek et al., 2014b); the difficulty and complexity of the tasks, leading to low solution rates and low numbers of problem trials within the practical limitations of investigative time-frames (Bowden and Jung-Beeman, 2003b; MacGregor and Cunningham, 2008; Batchelder and Alexander, 2012; Danek et al., 2016); and the memory advantage obtained for solutions arrived at by insight (Dominowski and Buyer, 2000; Danek et al., 2013) which rules out test-retest options (MacGregor and Cunningham, 2008).

**Figure 1:**
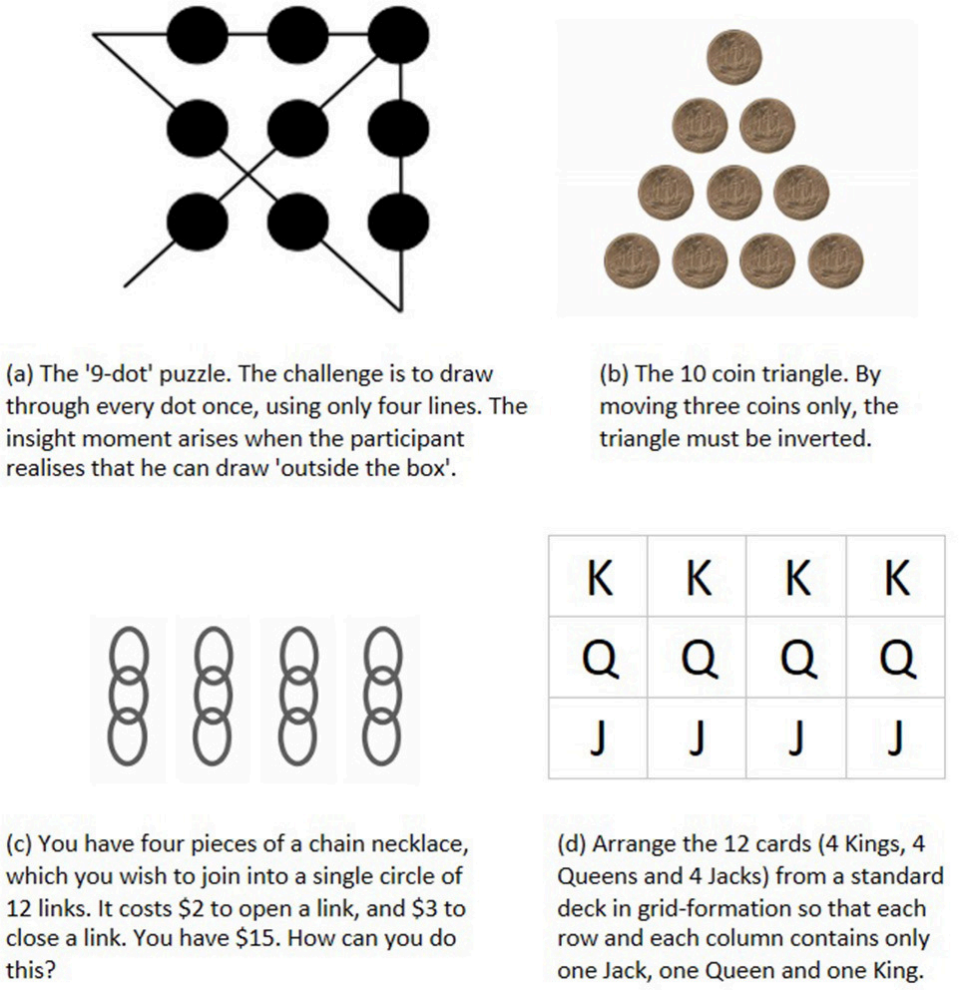
Classic brainteaser puzzles used to explore insight: see further Cunningham et al. (2009).

This last issue poses a particular problem for controlled, lab-based research, given that the solutions to so many of the classic riddle-style “insight problems” (e.g., the 9-dot problem, the reversed triangle of coins, the broken necklace challenge—Cunningham et al., 2009—see Figure 1) are now freely available on-line and in puzzle collections; this commonly leads to the need to discard trials due to familiarity with the puzzles (Öllinger et al., 2014; see also Danek et al., 2016).

Following attempts to increase the pool of test material in recent years, larger collections of calibrated problems do now exist (Chu and MacGregor, 2011): these have moved away from the classic “riddle-style” puzzles (Webb et al., 2016) and might include matchstick arithmetic problems (Knoblich et al., 1999), compound remote association problems (“CRA”—a variation of “Remote Association Test” (RAT) problems—Bowden and Jung-Beeman, 2003b), the “Car Park Game” (Jones, 2003), rebus puzzles (MacGregor and Cunningham, 2008), Bongard problems and “tricky series completion” problems (Batchelder and Alexander, 2012). Recently, magic tricks have been added to the list of available paradigms (Danek et al., 2014b).

### When is insight “insight”?

The use of a canonical set of “insight problems” to explore “Aha!” moments in the laboratory has led to a long-standing debate concerning the underlying cognitive mechanisms involved in their solution: specifically, whether an “Aha!” feeling is the result of “special” thought processes, or is merely an epiphenomenon arising from cognitive processes which are “business as usual” (for a review of this debate see Davidson, 1995; Bowden et al., 2005; Ohlsson, 2011; Gilhooly et al., 2015; Weisberg, 2015). One confounding issue which has hampered investigation of this question is the common assumption in many historical studies that “insight problems” are, *per se*, always solved with insight by every successful solver; in other words, that triggering insight is an inherent and objective property of the “insight problem” which unfailingly comes into play (Bowden and Jung-Beeman, 2007; Ohlsson, 2011; Öllinger et al., 2014). Crucially, as a result of this *a priori* assumption, no check was typically made as to whether the “Aha!” moment had actually been experienced in these trials, leading to a highly problematic circularity: “Insight problems are problems that require insight, and insight occurs when insight problems are solved” (Öllinger and Knoblich, 2009, p. 277; see also Danek et al., 2016; Webb et al., 2016). An early attempt (Weisberg, 2015; see Ash et al., 2009) to circumvent this problem by categorizing “insight problems” into “pure” problems (those that could only be solved with insight), “hybrid” problems (those that could be solved through insight and other methods) and “non-insight” problems (those which are always resolved through an analytical approach) nonetheless still requires that a subset of problems exists which infallibly trigger insight.

A critical flaw in this approach is that it overlooks the interactive nature of problem solving: successful solving arises from the interplay of problem and person, with each individual bringing a unique blend of knowledge, experience and cognitive approaches to bear upon it (Ash et al., 2009; Ohlsson, 2011). It is therefore entirely possible for a so-called “insight puzzle” to be solved through controlled, deliberate, systematic and evaluative means by some solvers—analytic “Type 2” thinking according to dual process theory (Evans and Stanovich, 2013; Sowden et al., 2015; Weisberg, 2015)—which is not thought to give rise to a characteristically strong emotional response, other than satisfaction at the job completed (Kounios and Beeman, 2014).

Others, however, may solve the same puzzle with a flash of inspiration that they could not predict, through processes operating below the threshold of their awareness, and will experience the impact of the “Aha!” moment. Much will depend on what each solver brings to the solving process: “each problem can be solved without insight if the initial problem representation is adequate and the appropriate heuristics are available” (Öllinger et al., 2014, p. 267), and this will vary from solver to solver according to their skill-set and experience. The presence or absence of insight thus resides in the solver's approach to solving the puzzle, not simply in the problem itself (Bowden and Jung-Beeman, 2007; Cunningham et al., 2009; Webb et al., 2016), and the categorization of “insight problem” stimuli as “pure” or “hybrid”, or “insight/non-insight” on the grounds of a hypothetical cognitive task analysis appears to be fundamentally flawed (Ash et al., 2009; Webb et al., 2016).

The purpose of insight research should not therefore be to develop a single theory which accounts for all solutions to “insight problems” arrived at by any manner under experimental conditions (Ohlsson, 2011), but to isolate those solutions which have evoked the phenomenological events specifically characteristic of an “Aha!” event, and to use these to explore the cognitive mechanisms underlying this experience (Webb et al., 2016). More contemporary studies have typically achieved this by collecting subjective feedback from trial participants as to whether they have actually experienced an “Aha!” moment at the point of solution (Bowden and Jung-Beeman, 2007; Kounios et al., 2008; Cranford and Moss, 2011; Jarosz et al., 2012; Danek et al., 2014b; Salvi et al., 2016b; Webb et al., 2016). This technique has been validated by a number of neuroimaging studies, which have empirically demonstrated meaningful differences between problems identified by participants as being solved with insight, or in a step-wise fashion (Zhao et al., 2013; Kounios and Beeman, 2014).

### Representational change theory

Notwithstanding this, it would be unhelpful to reject the term “insight problem” altogether, given that it is clear that some cognitive puzzles are more likely to trigger insight moments than others (Danek et al., 2014a), and indeed “insight problems” may operate along a continuum of efficacy (Webb et al., 2016). In particular, Representational Change Theory (“RCT”—Ohlsson et al., 1992; Knoblich et al., 1999; Ohlsson, 2011; Öllinger et al., 2014) suggests that especially effective insight-triggering puzzles use the solver's prior knowledge and expectations to deliberately induce a false conceptualization of the problem (Ovington et al., 2016), leading to self-imposed constraints which impede a solution. This can result in a feeling of “impasse”: the situation where the solver feels that they have explored all possible approaches to resolving the problem, and is now at a loss as to what to try next (Knoblich et al., 2001).

The moment of insight is argued to be the point at which the hindering constraint is suddenly removed, leading to a relaxation of the impasse and the rapid redefining of the problem space, followed by a swift solution. The initially incorrect reading of the problem—termed mental set by the Gestalt school (Wiley, 1998; Öllinger et al., 2008)—is argued to arise unavoidably and unconsciously from implicit assumptions or well-practiced procedures which are activated highly automatically (Ohlsson et al., 1992; Knoblich et al., 1999; DeYoung et al., 2008; Öllinger et al., 2008; Danek et al., 2014b; Patrick et al., 2015), making the less obvious, but correct, interpretation of the problem very unlikely to come to mind. It is the dropping of the incorrect assumptions, and disengagement from the outdated hypothesis, which is argued to allow progress to be made.

### Heterogeneous nature of insight puzzles and their mechanisms

It is thus widely acknowledged that “insight problem” solving involves some form of reconstructive change of the initial representation of the problem (Chronicle et al., 2004; Cunningham et al., 2009; Danek et al., 2014a); however, the precise mechanisms to achieve this reconstruction—and whether they are in any way “special”—remain unclear.

A number of theoretical models to explain this restructuring in classic insight puzzles, such as the 9-dot or the 8-coin puzzles, have been put forward: for example “elaboration, re-encoding or constraint relaxation” (Ohlsson et al., 1992); “opportunistic assimilation” (Seifert et al., 1995); “constraint relaxation and chunk decomposition” (Knoblich et al., 1999); “solution-recoding” (Chronicle et al., 2004); see further the reviews by Ash et al. (2009) and Batchelder and Alexander (2012). Nonetheless, since the formulation of these theories, a wider range of insight-triggering paradigms has been developed which on at least superficial grounds differ greatly in their appearance and the demands they make upon the solver (Bowden et al., 2005). It is therefore at least possible that the cognitive processes leading up to the moment of restructuring differ according to the specific puzzle parameters at play (Bowden and Jung-Beeman, 2007), making a single-process theory of restructuring difficult (Cunningham et al., 2009).

In a study comparing the relationships among a small range of diverse insight puzzles (classic “spatial” puzzles, RAT puzzles and rebus problems), Cunningham and colleagues identified the following characteristics of restructuring which they believed were displayed, to a greater or lesser extent, by each of their puzzle formats of interest (Cunningham et al., 2009). As predicted by RCT, some puzzles involved the need to overcome misdirection or the relaxation of automatically elicited constraints concerning the existing components of the puzzle or its spatial layout (Cunningham et al., 2009). However, in others, the primary difficulty appeared to lie in identifying what the eventual solution would look like, perhaps requiring the assimilation of extra incidental information, a sudden “figure-ground” reversal of perspective, or additional steps in order to hit upon the solution (Cunningham et al., 2009).

One methodological issue thus lies in how “well-defined” a problem type is (DeYoung et al., 2008; see also Simon, 1973; Davidson, 2003; Pretz et al., 2003; Hélie and Sun, 2010; Danek et al., 2016; Ovington et al., 2016; Webb et al., 2016). An ill-defined problem has no clear representation of the problem space in terms of key features such as the initial conceptualization of the challenge, the final goal state, and the mechanizable steps which need to be taken to achieve this goal. By contrast, “well-defined” problems may be tackled by controlled and systematic paradigmatic processes leading to steady progress toward a known target state (Smith, 2003; DeYoung et al., 2008), and better defined problems of this kind therefore lead less often to solution through insight (Webb et al., 2016).

Despite early attempts to categorize insight puzzles (e.g., as pure/hybrid) according to solving process (Ohlsson et al., 1992; Weisberg, 1995; Ansburg and Dominowski, 2000), the heterogeneous nature of the various problem collections therefore makes equivalence studies difficult (Weisberg, 1995; Cunningham et al., 2009), and this limits our understanding of the core components of problem solving with insight (Bowden and Jung-Beeman, 2003b; MacGregor and Cunningham, 2008). Attempts to find one single explanation of the cognitive processes leading to insight solution by pitting alternative theories against each other on a single puzzle type (e.g., Jones, 2003) may on this account be doomed: it is entirely possible that insight could arise from different interacting sets of preceding processes depending upon the context and the challenge inherent in the problem and that these processes may only imperfectly map onto these traditional problem type categories (Bowden and Jung-Beeman, 2007; Shen et al., 2016). A theoretical or computational model of “insight problem” solving which satisfactorily explains all facets and styles of insight challenge is therefore proving elusive (Ash et al., 2009; Batchelder and Alexander, 2012).

### Rapid solving and incubated problems

Equally vexed is the question of whether a period of impasse is always involved in insight problem-solving (as argued e.g., by Ohlsson et al., 1992), with some studies reporting that—even within puzzle type—solvers did not uniformly experience a period of impasse (Ash et al., 2012; Cranford and Moss, 2012; Danek et al., 2014a).

Indeed, studies have suggested that solvers can experience an instantaneous “Aha!” moment within seconds of the presentation of the puzzle. In a study of anagram solving, Novick and Sherman noted that “pop-out” solutions tended to be the first solution offered and to occur within 2 s of the presentation of the letters (Novick and Sherman, 2003). In trials of highly skilled anagram solvers, 47% of the solutions were reported to be immediate “pop-out” solutions, where the solver agreed that, “The solution came to mind suddenly, seemingly out of nowhere. I have no awareness of having done anything to try to get the answer.” By contrast 27% of solutions occurred with insight after a period of trying fruitless combinations; and 26% were generated incrementally by the recursive testing of morphemically probable combinations (non-insight search solutions).

Similarly, a study of RAT problems (Cranford and Moss, 2012), found that 171 out of 218 solutions arrived at with self-reported insight, under think-aloud conditions, were solved almost immediately, in a mean time of 7.1 s. These were categorized as “Immediate Insight” (II) moments; however, the authors also raised the possibility that the solution might simply have occurred so fast that it appeared sudden and surprising, without evoking the full phenomenological experience (Cranford and Moss, 2012; see also Topolinski and Reber, 2010b). Indeed, an fMRI study comparing II with Delayed Insight (DI) RAT solutions showed large differences in activation patterns for the two types of insight, suggesting that they may represent distinct solution processes (Cranford and Moss, 2011). For this reason, some later studies have excluded II solutions from their discussion, on the grounds that they may not reflect the full “Aha!” experience (e.g., Salvi et al., 2016a).

Conversely, the benefits of a period of incubation (non-conscious solving activity, or a period of respite away from the problem) in resolving problems which have reached impasse have been well-documented (see the meta-analytic review by Sio and Ormerod, 2009; also Ohlsson, 2011; Baird et al., 2012; Sio and Ormerod, 2015; Gilhooly, 2016), although the mechanisms which account for the facilitation of the solution (e.g., “unconscious work,” “intermittent work,” “beneficial forgetting”—Gilhooly, 2016) are as yet unclear. Incubation is clearly not always involved in insight problem resolution—though it was present as the second of Wallas' (1926) four stages of insight problem-solving (Sio and Ormerod, 2009)—and is rather seen as an ancillary feature, to be utilized where necessary (Gilhooly, 2016). Engaging in a diversionary activity with a low cognitive load appears to be most helpful (Sio and Ormerod, 2009), and many people report that the problem solution occurs to them when engaged in everyday activities such as walking, driving, or showering (Hill and Kemp, 2016; Ovington et al., 2018); a substantial number also report facilitation overnight, during their dreams or immediately upon waking (Ovington et al., 2018).

## Cryptic crosswords as potential triggers of insight

Cryptic (British-style) crosswords afford a unique opportunity to explore the mechanisms of insight and the issues highlighted above within an existing, readily available puzzle format. Devised in the mid 1920's (Connor, 2014), cryptic crosswords employ an extensive variety of highly ingenious puzzle mechanisms, many of which also draw on shared characteristics with a range of other types of “insight problem” (see review below). One puzzle may thus encapsulate a wide range of these mechanisms, presenting a compendium of heterogeneous insight challenges unrivaled by any other insight puzzle format. Studying cryptic crosswords may therefore enable us to understand better the antecedents, solving processes and key triggers of the insight moment.

### What are “cryptic crosswords”?

The nature of the cryptic crossword has been described in some detail in an earlier paper (Friedlander and Fine, 2016), but key aspects are highlighted again below. Example cryptic crossword clues, together with an explanation of the cryptic instructions for achieving the required solution, are set out in Boxes 1, 2, 4–6.

Box 1 **Illustration of cryptic clue mechanisms: misleading surface readings.**

**Clue 1(a) Active women iron some skirts and shirts (9)**—(Schulman, 1996, p. 309)

The definition is “Active women” = an obliquely phrased straight definition for FEMINISTS

The wordplay comprises: FE (iron, chemical symbol) + MINIS (plural form of a type of skirt, hence the word “some”) + TS (= plural of “T”, an abbreviation for “T-Shirt”)

The surface meaning is highly misleading; additionally, the interpretation of IRON relies on a linguistic ambiguity (homonym employing different part of speech - noun, not verb).

**Clue 1(b) Grown-up kid starts to gossip on aunt's Twitter (4)**

The definition is “Grown-up kid” = a misleading circumlocution for GOAT

The wordplay plays on the word “starts” (in the nounal sense of “leading letters,” not verbal sense of “begins”) as an acrostic indicator: “Gossip On Aunt's Twitter.”

**Clue 1(c) Scrub the cooker top and clean out (6)** - (Cleary, 1996, from the Guardian, No. 20248, 26 Jan 1995)

The definition is “Scrub” = CANCEL, a non-prototypical interpretation.

The wordplay is a complex anagram of “C” (= “the cooker top” i.e. its initial letter) + CLEAN. The anagram indicator is the word “OUT.”

An important secondary function of the wordplay is to guide the solver away from the required definition of the target word, and to strongly promote the more prototypical sense “Scrub = Clean” by contextual means (Cleary, 1996).

**Wordplay elements (Friedlander and Fine**, **2016****)**

The algebraic/programming nature of the cryptic clue means that wordplay components may be flexibly recombined or anagrammed to form new units, e.g.:

- A+B = C (FAT+HER = FATHER)
- rev(A) = B (TRAMS -> SMART)
- anag(A+B) = C (CAT+HAT = ATTACH)
•trunc(A) = B (CUTTER -> UTTER)

Clues usually contain an “indicator” identifying what type of transformation is required (Biddlecombe, 2009), but equally might be of a punning/novelty type (usually indicated by a question mark at the end of the clue).

Box 2 **Illustration of cryptic clue mechanisms: jokes and puns.**

**Clue 2(a) Frightened to death? (6,5)** - (Cleary, 1996)

Answer = SCARED STIFF, with a punning reference to “STIFF” = “corpse,” confirming the correctness of the solution.

**Clue 2(b) Discovered why electrical equipment was dangerous? (9)** - (Collingridge, 2010)

Answer = UNEARTHED (the latent secondary sense relates to electrical wiring)

**Clue 2(c) Yorkshire beauty queen, we hear, pulls the wool over one's eyes (8)** (“Orlando,” in Connor, 2011b)

Answer = MISLEADS. The pun (“Miss Leeds”) is indicated by a homophone indicator “we hear,” common in joke-style clues.

**Clue 2(d) A wicked thing? (6)** - (Aarons, 2015)

Answer = CANDLE. The clue relies on the two different homographic senses of the word “wicked.” Difficulty is heightened by the distinctly different pronunciation (/wik'id/; /wikt/) and by the non-prototypical sense of “wicked” which is required (= “possessing a wick”). As in most punning or riddle-style clues, the quirky or nonsensical nature of the answer is flagged by the use of a question mark, which serves as a clue-type indicator.

Unlike their “straight definition” American cousins, the challenge of the British-style cryptic crossword lies not in the obscurity of the vocabulary to be retrieved, but in the quasi-algebraic coded instructions which must be executed precisely in order to achieve the correct answer to the clue (Friedlander and Fine, 2016): see Box 1. Cryptic crossword clues usually comprise two elements: a straight definition, plus the cryptic instructions for assembling the required solution—the “wordplay” (Friedlander and Fine, 2016; Pham, 2016). It is not always obvious which part of the clue is fulfilling what role, and there is often no clear division between the two parts (Friedlander and Fine, 2016). Even the “definitional” element of the clue might be obliquely or whimsically referenced, consciously exploiting ambiguities such as grammatical form, phrasal semantics, homophones, synonyms, and roundabout expressions (Cleary, 1996; Aarons, 2015; Friedlander and Fine, 2016). The clue type also has to be identified and interpreted. All these factors mean that that cryptic crosswords are typically ill-defined in both problem conceptualization and solution methodology (Johnstone, 2001).

Each cryptic crossword clue is thus a tricky linguistic puzzle using non-literal interpretations of deconstructed clue components in a “truly slippery and fundamentally ambiguous” fashion (Aarons, 2012, p. 224), stretching the conventions of everyday speech at all levels of structure and context (Aarons, 2015). The misdirection is deliberate: the surface reading of the clue evokes our tacit knowledge of language to suggest a plausible, yet unhelpful, interpretation of the clue (the “red herring”), setting up a constraint which must be resolved for progress to be made (Aarons, 2015; Friedlander and Fine, 2016). Once accomplished, the “Aha!” experience is triggered: this is termed the “Penny Dropping Moment” or “PDM” by crossword solvers (Friedlander and Fine, 2016).

In this use of misdirection, cryptic crosswords are similar to magic tricks: in both areas, the practitioner exploits implicit assumptions of the audience which are activated highly automatically, either (in magic) because of long-term exposure to the natural laws governing everyday life, such as gravity (Danek et al., 2014b) or (in crosswords) because of a lifetime's parsing habits as a reader and interpreter of standard text (Schulman, 1996). The task of the setter, as for the magician, is to conceal the clue mechanism so subtly that the pathway is not readily detectable (Friedlander and Fine, 2016).

Once deconstructed in this manner, there is no requirement for the cryptic components to make further sense as a coherent whole: the beguilingly smooth surface reading of the clue is typically abandoned in favor of a *potpourri* of dissociated cryptic fragments, each serving a quite different purpose entirely ungoverned by word-order, grammatical or orthographic considerations (Pham, 2016). In this way cryptic crosswords can be seen as a type of “non-*bona fide* communication” (Aarons, 2015, p. 357): the solver understands that the normal rules of communication must be temporarily suspended (just as they are required to suspend disbelief at a magic show), and that the clue itself is simply a vehicle for the intellectual challenge of solving the clue.

### Range of cryptic clue challenges and parallels with other insight problems

Although there is general agreement that the clues have to be fairly constructed (i.e., unambiguously solvable), there are no hard-and-fast guidelines as to what the rules of engagement are (Aarons, 2015; Friedlander and Fine, 2016), leading to an almost infinite number of innovative ways to exploit the “versatile and quirky English language” (Connor, 2013). Nevertheless, there is some consensus over a number of basic mechanism types, and a range of “Teach-Yourself” primers exist (Friedlander and Fine, 2016: see also now the on-line solving channel - Anthony and Goodliffe vlog, n.d.). A brief review of the most striking parallels between a variety of insight puzzles and the mechanics of solving cryptic crosswords follows.

### Jokes and cryptic crosswords: deliberate misdirection

Individual differences in the ability to appreciate humor have been previously identified (Cunningham and Derks, 2005; Kozbelt and Nishioka, 2010; Dunbar et al., 2016) and cryptic crossword solvers appear to be particularly attuned to and to enjoy verbal ambiguity and wordplay. In a study involving solvers and non-solvers (Underwood et al., 1988) the strongest correlation associated with cryptic puzzle-solving was the frequency of incidentally elicited laughter during an experiment involving associative priming (e.g., “strawberry” priming “traffic” through the unpresented word “jam”).

Linguistic jokes share many characteristics with cryptic crosswords, including deliberate misdirection (Aarons, 2015), and—although only rarely used as such in the lab—jokes have been identified as a type of insight puzzle (Gick and Lockhart, 1995; Ramachandran, 1998; Robertson, 2001; Kounios and Jung-Beeman, 2009; Kozbelt and Nishioka, 2010; Amir et al., 2015) on the basis of the suddenness and rapidity of the solution, the lack of “feeling-of-warmth,” the pleasant feelings evoked at the moment of understanding, and the feeling of certainty in the correctness of the solution. A punning joke is typically based on two alternative interpretations of a scripted feed-line, which are both plausible in some sense, however absurd, “until the punchline, which highlights the initially less obvious one, and reveals the other to be a dummy, designed intentionally to mislead the listener” (Aarons, 2015, p. 352).

Working in a parallel tradition to that of psychological insight studies, linguistic humor studies have long explored the operation of jokes in the context of a two-stage process of “Incongruity-Resolution” (for a review see Forabosco, 2008), which shares many points of similarity with RCT. “Incongruity-Resolution” proposes that the expectations of the joke's audience are deliberately manipulated to predict a sensible, but incorrect outcome, making the actual punchline initially unexpected or incongruous (the “surprise” phase). In the second phase (termed “coherence”), the listener then engages in a rapid form of problem-solving in order to revisit and resolve the incongruity, enabling the punchline to make plausible sense once it has been reconciled with an amusing and perhaps off-beat alternative interpretation of the original joke setting (Suls, 1972; Bartolo et al., 2006; Forabosco, 2008; Hurley et al., 2011; Canestrari and Bianchi, 2012). In other words, they must backtrack to search for an implicit constraint in their interpretation of the joke wording, which can be relaxed sufficiently to accommodate both the joke setting and its punchline within a revised interpretative structure (Suls, 1972; Navon, 1988). This process takes only a short time: there is an inverted relationship between speed of appreciation and funniness ratings (Cunningham and Derks, 2005; Kozbelt and Nishioka, 2010), and a joke falls flat if the explanation is too labored (Kozbelt and Nishioka, 2010).

If interpreted literally, the initially less dominant meaning (“latent content”—Kozbelt and Nishioka, 2010; Erdelyi, 2014) underpinning the correct interpretation of the punchline is often inappropriate, impossible or surreal: an “as if” resolution (Navon, 1988; Amir et al., 2015) which is “seemingly appropriate but virtually inappropriate” (Navon, 1988, p. 210) and—as for cryptic crosswords and magic tricks—functions “only on account of a willing suspension of disbelief” (Attardo et al., 2002, p. 5). It is at this point that we experience the emotional payback, as we “get” the joke, with the sudden, absurd resolution eliciting laughter; recent studies have begun to explore the neural correlates of these humorous insight moments (Amir et al., 2015; Chan, 2016).

The workings of this mechanism are exemplified in the following joke:

‘*So, I bought some animal crackers, and the box said:*

“*Do not consume if the seal is broken”…*’ (attrib. Brian Kiley)

Here, the listener is primed to interpret the term “seal” in terms of the intact packaging containing the foodstuff. The punchline seems incongruously out of place given that a joke is ostensibly being recounted: it appears to be a banal repetition of standard wording commonly found on packaged goods, and is not inherently amusing. The feeling of “missing something”—that “*nagging* sort of anxiety when you sense that something is funny-huh” (Hurley et al., 2011, p. 79) evokes an uncomfortable state of incongruity akin to cognitive dissonance (Festinger, 1957; Forabosco, 2008; Yim, 2016), and this discomfort will provide the motivational drive to reconcile or reduce the perceived inconsistency by reassessing the initial interpretation of the joke setting. It is only upon reinterpreting the word “seal” (in the context of “animal crackers”) that the alternative and nonsensical latent content of the joke emerges: that the crackers should not be eaten if the seal biscuit is broken.

Similarly, the cryptic crossword clue at Box 2a leads initially to a deceptively straightforward solution (“Scared stiff”), which perhaps only subsequently reveals the underlying pun “Stiff—> Corpse—> Frightened to death,” confirming the accuracy of the solution.

Fundamental to punning humor of this nature is the concept of “bisociation”—the perceiving of a situation in two incompatible frames of reference (Koestler, 1964; Dienhart, 1999; Canestrari and Bianchi, 2012). Following this account, ambiguous phonetic forms such as homophones, homonyms, and polysemes can act as triggers which abruptly switch the listener from one semantic script (e.g., “seal = box packaging”) to another (e.g., “seal = biscuit shape”). Koestler sees this as a sudden “Gestalt” reversal (Koestler, 1964).

Key to the workings of the joke or crossword clue is the initial concealment of the alternative meaning; and indeed it is a general feature of insight puzzles that the solution typically involves a statistically infrequent response, such as an unusual use for an object, or a less familiar, less dominant meaning for a word or phrase (Dominowski, 1995). So, for example, the cryptic crossword clue at Box 2b requires the solver to recognize that a potential solution word (“unearthed”), in its prototypical sense of “discovered,” has a second, non-intuitive but highly appropriate role to play in the clue (“without an earth wire”).

The cryptic crossword solver is thus often gulled into a readily available, but false interpretation of the clue setting (the “surface reading”) based on a *prima facie* interpretation of everyday linguistic rules, ambiguous phonetic forms, learned phraseological conventions, and context. This approach leads initially to nagging puzzlement, impasse and cognitive dissonance, since the original interpretation cannot be made to yield the desired answer (the solver is “missing something”). This provides the motivation to detect and explore alternative interpretations (some perhaps fruitlessly) in order to arrive at the moment of insight. As with jokes, the cryptic crossword's “pay-off” (the final understanding of the clue) arrives when the original constraints are abruptly overturned in favor of a switch to an alternative, non-intuitive reading of the cryptic elements—often leading to surprise, laughter and the delight of the PDM (Aarons, 2015). No matter how lengthy and difficult this problem-solving phase has been, the clue is typically processed rapidly once the constraint is cracked (Topolinski and Reber, 2010a).

### Rebus puzzles and cryptic crosswords: reinterpretation of visual/spatial elements

Although many cryptic crossword clues rely heavily on punning misdirection, many also employ clue mechanisms which indicate that letters or letter blocks must be transposed, reversed, removed, substituted, extracted from a sequence or read as an acrostic (Aarons, 2015). In these clues, the elements providing the wordplay fodder must be decontextualized from the natural surface reading, either abandoning meaning altogether, or taking on new meaning of their own. Once these problem-irrelevant “chunks” have been decomposed (Knoblich et al., 1999) the components are redeployed in quasi-algebraic fashion to form new units answering to the clue definition (Friedlander and Fine, 2016): see further Box 1.

One clue type of this nature is the “charade”: a type of riddle in which the whole word is hinted at enigmatically by reference to its component syllables (Chambers, 2014). In this process, cryptic crosswords may not observe morphological rules: for example, the word “*discourage”* would be segmented linguistically as “*dis-courage*,” but in a cryptic crossword might be clued, as “*Di (girl's name)* + *scour* + *age*” (Aarons, 2015). See further clues 1(a) and 4(f) in Boxes 1, 4.

Similarly, rebus puzzles rely on the manipulation of words and word fragments to suggest common phrases which fit the clues displayed in a “word-picture.” Common rebus types involve charades, the interpretation of the spatial locations of words in relation to each other, typographical trends (letter size growing, decreasing), font size or color (capitalization etc.), numbers, and letters as words (MacGregor and Cunningham, 2008; Salvi et al., 2016b): see examples in Box 3. Rebus puzzles are also examples of ill-defined problems (Salvi et al., 2016b): the mechanisms for achieving the problem solution are unclear to the solver, who may have to try multiple strategies before hitting upon a productive approach. As with cryptic crosswords, the solver has to relax the ingrained rules of reading in order to overcome their tacit understanding of word-form and contextual interpretation and to achieve a restructuring of the problem space (Salvi et al., 2016b). For this reason, they are likely to trigger the insight experience (MacGregor and Cunningham, 2008; Salvi et al., 2016b).

Box 3 **Rebus puzzles.**

**3(a) poPPd** (MacGregor and Cunningham, 2008)

Solution: “Two peas in a pod”: auditory pun on “P” = “pea,” together with spatial location of the letters inside the word “pod.”

**3(b) TIMING TIM ING** (Smith and Blankenship, 1989)

Solution: “Split second timing”: the second instance of “timing” is split into two parts.

**3(c) M CE /M CE /M CE** (Salvi et al., 2016b)

Solution: “Three Blind Mice”: the mice have no “I”s (eyes)

**3(d) R. P. I**. (MacGregor and Cunningham, 2009)

Solution: “A grave error” (it should have been written as R.I.P.)

Rebus puzzles typically rely on the literal and quirky interpretation of encrypted elements and their spatial arrangement, which are interpreted as part of the solution (MacGregor and Cunningham, 2008). In the British TV programme “*Catchphrase*,” which was based upon the solving of pictorially displayed rebus-type puzzles, the host, Roy Walker, used the tag line “Say what you see” in order to prompt contestants to find the solution (Wikipedia, 2017b). This is precisely the approach needed by a number of the rebus-style cryptic crossword clues in Box 4 which use highly inventive gimmicks to cryptically represent the solution word (clues 4 b-e).

Box 4 **Illustration of cryptic clue mechanisms: rebus-like components.**

**Clue 4(a): Player with only one leg? (4)** (Guardian Crossword No. 25351, by Tramp; 17 June 2011)

Answer = IPOD, a type of music player.

The clue works by comic analogy to “TRIPOD,” with the letter “I” standing in for the numeral “one.” This is very similar to the rebus puzzle at Box 3a.

**Clue 4(b): Must've? (5,7,2,3,3)** (Guardian Crossword No. 25351, by Tramp; 17 June 2011)

Answer = THINK OUTSIDE OF THE BOX.

Wordplay: MUSE [think] outside of TV [“the box”] - a rebus-like construction, also telling the solver what he must literally do to solve the clue. The punctuation is a highly distracting feature.

**Clue 4(c): Part of it 'it an iceberg (7)** - (Moorey, 2009)

Answer = TITANIC.

Wordplay: substring(A+B+C+D) leading to a hidden word, indicated by the instruction “Part of.” The Titanic did indeed hit an iceberg, making this an “&Lit” (or “all-in-one”) clue: the clue as a whole functions as both the definition and the wordplay (Manley, 2014; Aarons, 2015).

**Clue 4(d): GEGS (9,4)** - (A well-known but unattributed clue, see Aarons, 2015).

Answer = SCRAMBLED EGGS. There is no guidance in the clue: the solver must literally “say what they see.” Compare the rebus examples 3(b) and 3(c) in Box 3 above.

**Clue 4(e): H,I,J,K,L,M,N,O (5)** - (Another old chestnut of uncertain provenance, see Aarons, 2015)

Answer = WATER. Wordplay: “H to O”, if spoken aloud, sounds like H_2_O.

**Clue 4(f): Somewhat swollen condition of female diving bird? (9) -** Times 24451, Feb 3rd 2010

Answer = PUFFINESS = “Somewhat swollen condition”

Wordplay = a quirky charade of PUFFIN + “-ESS” suffix, often indicative of a female in an animal species (e.g., “lioness”).

### Anagrams and cryptic crosswords: dechunking, pattern detection, and misdirection

Anagrams have been routinely used in investigations of insight (for a review, see Ellis et al., 2011)—both for anagram solving (e.g., Novick and Sherman, 2003; Kounios et al., 2008; Salvi et al., 2016a) and through the use of a paradigm requiring a simple judgment as to whether the anagram was solvable or not, in order to explore “feelings of warmth” and solution speed (e.g., Novick and Sherman, 2003; Topolinski and Reber, 2010b).

Studies of anagram solution have consistently reported that solvers approach anagram problems using two different strategies (e.g., Novick and Sherman, 2003; Kounios et al., 2008; Ellis et al., 2011; Salvi et al., 2016a): a search methodology, using a process of serially testing out and rejecting solutions based on morphemically probable letter combinations; and “pop-out” solutions (Novick and Sherman, 2003) whereby the solution bursts suddenly into consciousness without apparent work, often almost instantaneously. EEG research has demonstrated that self-reports distinguishing between “pop-out” and search anagram solving are reliably accurate (Kounios et al., 2008); this study also provides evidence that individual differences determine the solver's preferred strategy, and that different patterns of brain activity are associated with the two approaches.

It is well-established that structural features of the letter stimuli which are to be anagrammed (such as whether they are pronounceable, or form a real word in their own right) affect the difficulty and solution times of the puzzle. Thus, ZELBA or OARLY should be more difficult to resolve than HNWEI or AOSLR; and HEART should be more difficult to unscramble than THREA (Dominowski, 1969; Novick and Sherman, 2008; Ellis and Reingold, 2014; for a review see Topolinski et al., 2016). Dominowski suggests that the pronounceability of the letters leads solvers to deal with them as a unit rather than as a letter-sequence (Dominowski, 1969): in other words, that familiarity with the letter patterns sets up an obstacle to solution by accessing automatically stored “chunks” of data which will be inappropriate to the solution (cf. Knoblich et al., 1999). It is the decomposing of these chunks into component letters which paves the way to the solution.

Anagram clues are a staple of cryptic crosswords (Upadhyay, 2008b; Aarons, 2015, p. 371), being formed of the letters to be anagrammed (the “fodder”), an anagram indicator and the definition of the resulting word (see Box 5). The letter fodder is typically concealed in misleading word units, which will be unhelpful to the anagram solution as indicated above; for this reason, many solvers will write out the letter-fodder in a random arrangement (such as a circle), in order to try to break up the prior associations and allow new patterns to form (Johnstone, 2001—see Box 5). However, difficulty can also be heightened by misdirection in the surface reading and by heavy disguise of the anagram indicator.

Box 5 **Illustration of cryptic clue mechanisms: Anagram clues.**

**5(a) Tube taken to theatre for three-act play (8)** (Aarons, 2015, p. 371)

ANSWER = CATHETER (= “Tube taken to theatre”).

Letter fodder = THREE-ACT; anagram indicator = “PLAY”.

There is heavy misdirection drawing the solver away from the required medical context and into theatrical performance and the “London Underground” (the “Tube”).

**5(b) Doctor Watson's kit - or bits of modern office furniture (12)** (Biddlecombe, 2009)

ANSWER = WORKSTATIONS (”bits of modern office furniture”)

Letter fodder = WATSON'S KIT OR; anagram-indicator = “Doctor”

Misleading disguise of anagram indicator in the name “Doctor Watson”, making the parsing of the clue unclear.

**5(c) Find rare new frequencies beyond the visible range (8)** (Johnstone, 2001, p. 70)

ANSWER = INFRARED (”frequencies beyond the visible range”)

Letter fodder = FIND RARE; anagram indicator = NEW

Johnstone points out that solvers often write out candidate letters as shown below, in order to facilitate the solving process:

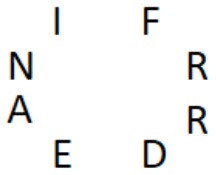

### Remote association puzzles and cryptic crosswords: spreading activation

The Remote Associates Test (RAT), originally developed as a test of creativity (Mednick, 1962), has been refined and updated on a number of occasions, resulting in several sets of test materials [Functional Remote Associates Test (FRAT) (Worthen and Clark, 1971); Compound Remote Associates (CRA) (Bowden and Jung-Beeman, 2003b)], and has been translated into a number of languages (Salvi et al., 2016b). The task challenge is for the participant to consider a triad of apparently unconnected words (e.g., *Cottage, Swiss, Cake*) and to come up with a fourth word (here *Cheese*) which is related to all three through some type of associative connective link.

Although no longer commonly used as a test of creativity *per se* (Salvi et al., 2016b), RAT are frequently used to study facets of creative problem-solving such as insight (Bowden et al., 2005; MacGregor and Cunningham, 2008; Cranford and Moss, 2012; Jarosz et al., 2012; Chein and Weisberg, 2014; Salvi et al., 2015; Webb et al., 2016), incubation effects (Smith and Blankenship, 1991; Cai et al., 2009; Sio and Ormerod, 2015), and fixedness upon the wrong solution (Smith and Blankenship, 1989, 1991).

RAT puzzles are thought to operate through a serendipitous spreading neuronal network (Collins and Loftus, 1975) akin to three ripples, whereby each triad member simultaneously but independently activates a retrieval search of semantic memory (Smith et al., 2012; Kenett et al., 2014; Olteteanu and Falomir, 2015). This global search operates as a multiple constraint problem, each cue word indicating a different attribute of the target word to be satisfied; the solution is arrived at by confluence of the ripples upon a jointly shared node (Gupta et al., 2012; Smith et al., 2013).

Alternatively, participants can adopt a more controlled generate-and-test strategy by considering just one of the three cues at a time, and testing out candidate solutions against each constraint for suitability, to ensure all requirements are met (Bowden and Jung-Beeman, 2007; Smith et al., 2013). This type of analytic, step-wise process is associated with lower insight ratings and different patterns of neural activity and eye movements when compared to sudden, non-methodical solutions (Bowden and Jung-Beeman, 2003a, 2007; Subramaniam et al., 2009; Cranford and Moss, 2012; Salvi et al., 2016b; Webb et al., 2016).

Impasse in solving RAT puzzles can arise from a fixation upon incorrect words, particularly those which are closely associated, syntactically or semantically, with one or more of the target words, and which therefore spring easily to mind (Harkins, 2006; Gupta et al., 2012). This blocks access to more remotely associated words needed for the solution (Gupta et al., 2012). Indeed, fixation in RAT problem-solving can be deliberately induced by priming commonplace associations which are unhelpful to the correct solution of the problem (Smith and Blankenship, 1991).

Consequently, one factor leading to higher performance on RAT puzzles is the ability to avoid a bias toward high-frequency candidate answers, thus allowing more remotely associated possibilities to be accessed (Gupta et al., 2012). This accords well with Mednick's conceptualization of an uncreative person as one who possesses a “steep associative hierarchy” containing an initially high number of stereotypical responses which rapidly tail off. By contrast, the highly creative individual will possess a “flat associative hierarchy” containing many more items, and fewer stereotypical responses (Mednick, 1962, p. 223). Creative individuals are thus argued to possess more associative links, leading to a more complex and less rigid lexical network (Gruszka and Necka, 2002; Kenett et al., 2014).

In general terms, RAT puzzles pose a similar challenge to the “definition” in cryptic crosswords, which may reference the target word with considerable concealment. In many cases, the sense required will not be the dominant association, but a secondary meaning (sometimes quite obscure) which will come much less readily to mind, and fixation upon the wrong sense is often deliberately induced by contextual means (Cleary, 1996—see Box 1c). Breaking free from the stereotypical interpretation in order to consider a range of potentially remote synonym options is therefore key to lighting upon the correct solution (cf. Dominowski, 1995).

Even closer to the format of the RAT puzzle, however, is the “double definition” clue (Biddlecombe, 2009; Connor, 2011a; Aarons, 2015), whereby the solver is presented with two words, both of which can be defined by the same polysemic or homographic solution word (Aarons, 2015; Pham, 2016). Occasionally, triad cryptic definitions (or even quadruple/quintuple) are also found (Connor, 2011a—see Box 6). As in jokes, double definition clues operate through “bisociation” and an unexpected pay-off: “the fun of seeing two disparate concepts suddenly become one” (Connor, 2011a).

Box 6 **Illustration of cryptic “double definition” clues: RAT-like mechanism.**

**Clue 6(a): Tea shop (5)** (Biddlecombe, 2009, attributed to Azed)

Answer = GRASS.

Synonym 1: “Tea” = slang for “marijuana” = GRASS

Synonym 2: “Shop” = slang for “betray to the police” = GRASS. “Shop” has to be taken as a verb in this meaning, in contrast to the nounal function in the clue itself.

**Clue 6(b): Savings book (7)** (Aarons, 2015, p. 365)

Answer = RESERVE.

Synonym 1: “Savings” = a RESERVE of money

Synonym 2: “book” = to RESERVE (a table etc.): again verbal (solution) rather than nounal (clue)

**Clue 6(c): Quits flat (4)** (Connor, 2011a, by Rufus)

Answer = EVEN

Synonym 1: “Quits” = “neither owing, nor owed” = EVEN: adjective, not verb

Synonym 2: “Flat” = “level” = EVEN: adjective, not noun

**Clue 6(d): Left red wine in harbour (4)** (Biddlecombe, 2009; Aarons, 2015, p. 366)

Answer = PORT, a triple-definition

Synonym 1: “Left” = “on PORT side”: adjective, not verb

Synonym 2: “Red wine” = fortified PORT wine

Synonym 3: “Harbour” = PORT

**Clue 6(e) Soldier even fixed uniform (7) -** Daily Telegraph 28392

Answer = REGULAR, a quadruple definition with a misleading military surface reading

Synonym 1: “Soldier” = REGULAR (i.e. member of permanent forces)

Synonym 2: “even” = “level” = REGULAR (adjective, not adverb)

Synonym 3: “fixed” = “at set intervals” = REGULAR (adjective, not verb)

Synonym 4: “Uniform” = “unvarying” = REGULAR (adjective, not noun)

Although the mechanism illustrated in Box 6 is very similar to that of RAT puzzles (“What one word links the following words?”), cryptic double definitions present extra difficulties, introducing elements of misdirection which are generally absent in RATs. First, in a dyad pairing, the two words are typically selected to form a familiar but unhelpful phrase with meaning of its own (e.g., 6(a) “tea shop”), creating a distracting red herring (Connor, 2011a). This automatically triggered impasse must be resolved by decomposing the unhelpful “chunked” phrase into its component features, allowing for an alternative parsing of the problem elements (Knoblich et al., 1999). Secondly, at least one of the words is usually “multicategorical,” meaning that it can used as different parts of speech in each of the clue and the solution (Aarons, 2015). Finally, the solver must identify the “double definition” mechanism unaided, since there is no clue-type indicator for this class (Upadhyay, 2008a). For all these reasons, double definitions can be one of the hardest clue types to crack (Connor, 2011a), requiring multiple constraining misconceptions about the meaning, form and function of the clue elements to be resolved.

### Advanced cryptic crosswords

So far, this article has only considered cryptic clues which might appear in daily “block-style” cryptic puzzles (Friedlander and Fine, 2016). However, a second type of cryptic crossword—advanced cryptics—also exists, which raises the difficulty still further (Friedlander and Fine, 2016). Advanced cryptic crosswords are found in weekend newspapers and some magazines, and the grids generally use bars rather than blocked grids (Friedlander and Fine, 2016). Of these, the *Listener Crossword* is the most notoriously difficult, employing a high degree of clue mechanism concealment, obscure vocabulary, grids of startling originality and a thematic challenge, often involving a number of tricky lateral thinking steps on the basis of minimal guidance (Listener Editorial Team, 2013; Alberich, n.d.). Solvers submit weekly solutions for the distinction of appearing on an annual roll of honor, but few achieve an all-correct year (Friedlander and Fine, 2016). The *Magpie*,^1^ a monthly specialist magazine with five highly challenging advanced cryptic crosswords (and one mathematical puzzle) per issue, runs a similar all correct/roll of honor system, and is broadly of *Listener* standard (Friedlander and Fine, 2016).

It is difficult to pigeon-hole the challenges set by advanced cryptics: there is an acute thirst for originality among the aficionados of these puzzles which drives setters to produce ever more creative designs, mechanisms and themes which “require original thinking by the solver over and over again” (Anthony, 2015), and annual awards for the most admired crossword in the *Magpie* and *Listener* series are presented to setters on the basis of solver recommendation (e.g., the *Listener* “Ascot Gold Cup^2^).” However, two particularly prominent sources of challenge are described below.

### Thematic challenge: acquisition of incidental hints

Many advanced cryptic puzzles contain a thematic challenge, lending extra difficulty to the puzzle. In one common approach, a number of thematically related entries may have no clue, requiring the solver to deduce the answers gradually from cross-checking letters, as the grid is populated. Additionally, entire areas of the grid—such as the complete perimeter—may need to be completed with thematically relevant items or messages. In other puzzles, letter sequences spelling out thematic material may be concealed in the grid (for example on the diagonals), requiring the solver to find and highlight them through a “wordsearch” process (Alberich, n.d.).

Thematic puzzles rely upon the solver's ability to make cross-connections between seemingly disparate items drawn from unpredictable and often obscure fields of knowledge: in this they share similarities with lateral thinking quizzes such as BBC2's *Only Connect* and BBC Radio 4's *Round Britain Quiz* (Connor, 2016). Once again, the problem space is ill-defined: the nature of the connection, the goal state and the pathway to achieve coherence are all unspecified.

In order to solve these puzzles, solvers have to accumulate incidental information along the way: hints in the title or preamble might point obliquely to the theme; suggestive word fragments might appear in the grid, and thematic material might be gradually spelled out by other means—such as corrections to misprints in the clues. The PDM comes at the instant when all the disparate pieces of information suddenly come together to make sense. It is therefore comparatively rare for the theme to be deduced from the start (indeed this element of the puzzle is often termed the “endgame”): the solver must be able to tolerate—or even enjoy—the sensation of working for some time with unclear goals and incomplete, potentially conflicting and imprecise data. This may imply that advanced cryptic solvers tend toward personality traits such as a low “Need for Closure”—the desire for definite knowledge and resolution of an issue (Webster and Kruglanski, 1994); and a high “Tolerance of Ambiguity”—the perceiving of ambiguous situations as desirable, challenging, and interesting (Furnham, 1994; Zenasni et al., 2008). Earlier research (Friedlander and Fine, 2016) has also found that cryptic crossword solvers generally have a high “Need for Cognition,” relating to a person's tendency to seek out, engage in and enjoy effortful thinking (see Cacioppo et al., 1984; Furnham and Thorne, 2013; Von Stumm and Ackerman, 2013).

**Figure 2:**
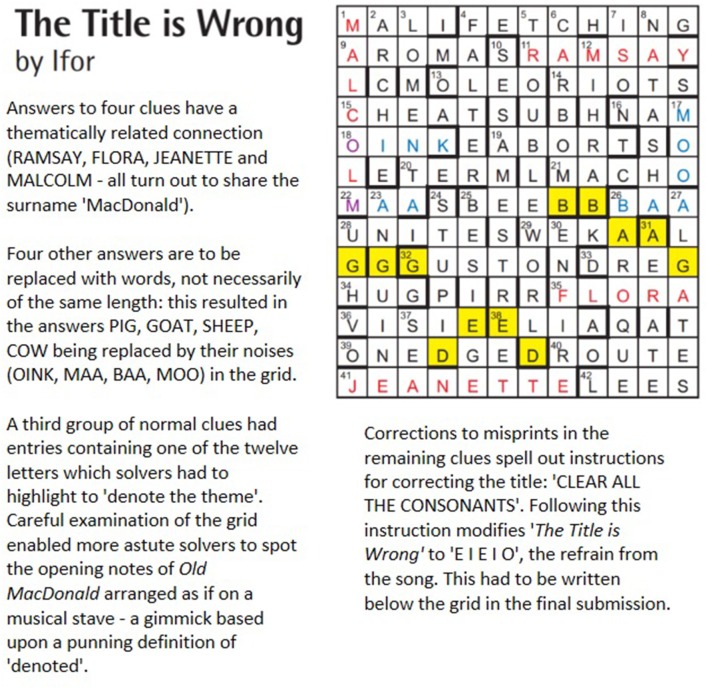
Magpie crossword issue 130.4 (Ifor, 2013).

An example of a thematic cryptic crossword challenge is shown in Figure 2. Here the well-known children's song “Old MacDonald Had a Farm” is used as a source of thematic material: “the super-familiar hiding under a thick cloak of obscurity, waiting to reward the determined solver with a PDM that feels like a surprise from an old friend” (Editorial Notes, 2013, p. 10).

Given the richness of the thematic material in this puzzle, which is expressed through multiple different devices (MacDonalds, animal noises, EIEIO title and the notation in the grid), it is likely that solvers experienced a number of PDMs—a series of mini “insight moments”—en route to a final solution. Some PDMs would almost certainly have come out of the blue: in particular, the concealed instruction to correct the title by deleting consonants “hides in a simple statement of fact a truly surprising vowel-only ‘correct’ title that nobody could possibly have seen coming” (Editorial Notes, 2013, p. 10). The finding of the tune proved trickier:

“*The common experience was an initial search (often for “MacDonald”), followed by some confusion, followed by careful examination of the letters in the appropriate area, followed maybe by re-reading the preamble, combined with spotting some suspect letter duplications … in other words, a penny that did drop, but did it slowly”* (Editorial Notes, 2013, p. 10).

As with RAT puzzles, thematic challenges appear to operate through a ripple of spreading activation (Collins and Loftus, 1975). Each “clue to coherence” (Bowers et al., 1990) embodies a different attribute of the target connection to be made; when these unconscious activations achieve confluence, the pattern emerges quite suddenly into consciousness, leading to the perception of coherence, and the PDM (a process described as “intuitive guiding”—Bowers et al., 1990). Individual differences will again arise in the speed, complexity and gradient of the available interassociative connections (Bowers et al., 1990; Gruszka and Necka, 2002; Smith et al., 2012; Kenett et al., 2014).

Individual differences in the ability to assimilate chance hints may also be relevant: as Louis Pasteur famously remarked of his ostensibly fortuitous scientific discoveries, “Chance favors only the prepared mind” (Lecture, University of Lille, 7 December 1854–Seifert et al., 1995). “Opportunistic assimilation” (Seifert et al., 1995; Sio and Ormerod, 2015) refers to the ability to absorb new and serendipitously presented information, and to allow these additional jigsaw pieces to resolve or reframe one's understanding of a problem which has previously reached impasse. Much may depend on the initial preparation stage in which the solver becomes attuned to salient or important features they have already noted (Seifert et al., 1995; Ormerod et al., 2002) which they maintain at a heightened level of activation, leading to priming effects (Sio and Ormerod, 2015). Although potentially experiencing a number of failures and false leads in the process (Ormerod et al., 2002), progress is then made when the solver becomes intrigued by further patterns or anomalies (Kolodner and Wills, 1996), or stumbles across other relevant information (Weisberg, 2006) during completion of the grid.

The process is well-illustrated by the editorial feedback on *Magpie* 151/2 “*Five-a-side (on Tour)*” by Wan, which was themed around a subset of the 72 names of French scientists, engineers and mathematicians engraved on the Eiffel Tower (five from each side):

“*In solving terms, there was a single critical, and memorable, moment of realization when the set of names suddenly made sense. This was normally preceded by a number of less memorable moments of thinking that there was some other reason for grouping, by nationality, or by specialization, or by university affiliation, or whatever. All the false trails had some value, because you were always going to be alert to French scientists or engineers once a few showed up. The feeling was of constant small steps forward, always with some difficulty, but never with that feeling of brick-wall despair that can accompany certain thematic endgames.”* (Editorial Notes, 2015, p. 9).

Individual differences in openness to experience and sensitivity to external stimuli could be relevant in these contexts, regulating the degree to which a person inhibits or remains subconsciously receptive to ostensibly incidental information (Laughlin, 1967; Carson et al., 2003; Simonton, 2003; Weisberg, 2006; Carson, 2010; Russ and Dillon, 2011). A reduced tendency to pre-filter extraneous information as irrelevant (i.e., reduced latent inhibition) may enhance the ability to make lateral associations, and has been associated with both psychometrically and behaviorally assessed creativity, openness to experience, and richer, more diverse associative networks (Simonton, 2003; Carson, 2010).

### Spatial or transformational challenges: reconceptualizing the layout

An additional source of difficulty in many advanced cryptic crosswords lies in the transformation of some elements. For example, some or all of the answers might need to be encoded or otherwise thematically altered before being entered in the grid. As in American-style “variety puzzles,” such as those appearing periodically in the Sunday edition of the NY Times (Wikipedia, 2017a), this might involve anagramming, reversing or curtailing entries (resulting in non-words in the grid); but more complex adjustments might also be required. For example the solver might deduce that all overlong items, such as APHID (to fit a grid space of 3) and CHINWAG (to fit 5), might need to be entered using Greek characters to replace the English names for the Greek alphabet (i.e., AΦD and *X*NWAG Alberich, n.d.). Or all entries might need to be encoded using a Playfair cipher, with the keyword to be deduced (Upadhyay, 2015). Once again, the problem space is ill-defined: the solver has to assimilate key hints or salient features as the puzzle progresses in order to deduce what adjustments need to be made, and may pursue a number of false leads before hitting upon the correct solution. Meanwhile, the completion of the grid is made much harder by the absence of securely confirmed cross-checking letters while the entry mechanism remains unresolved.

**Figure 3:**
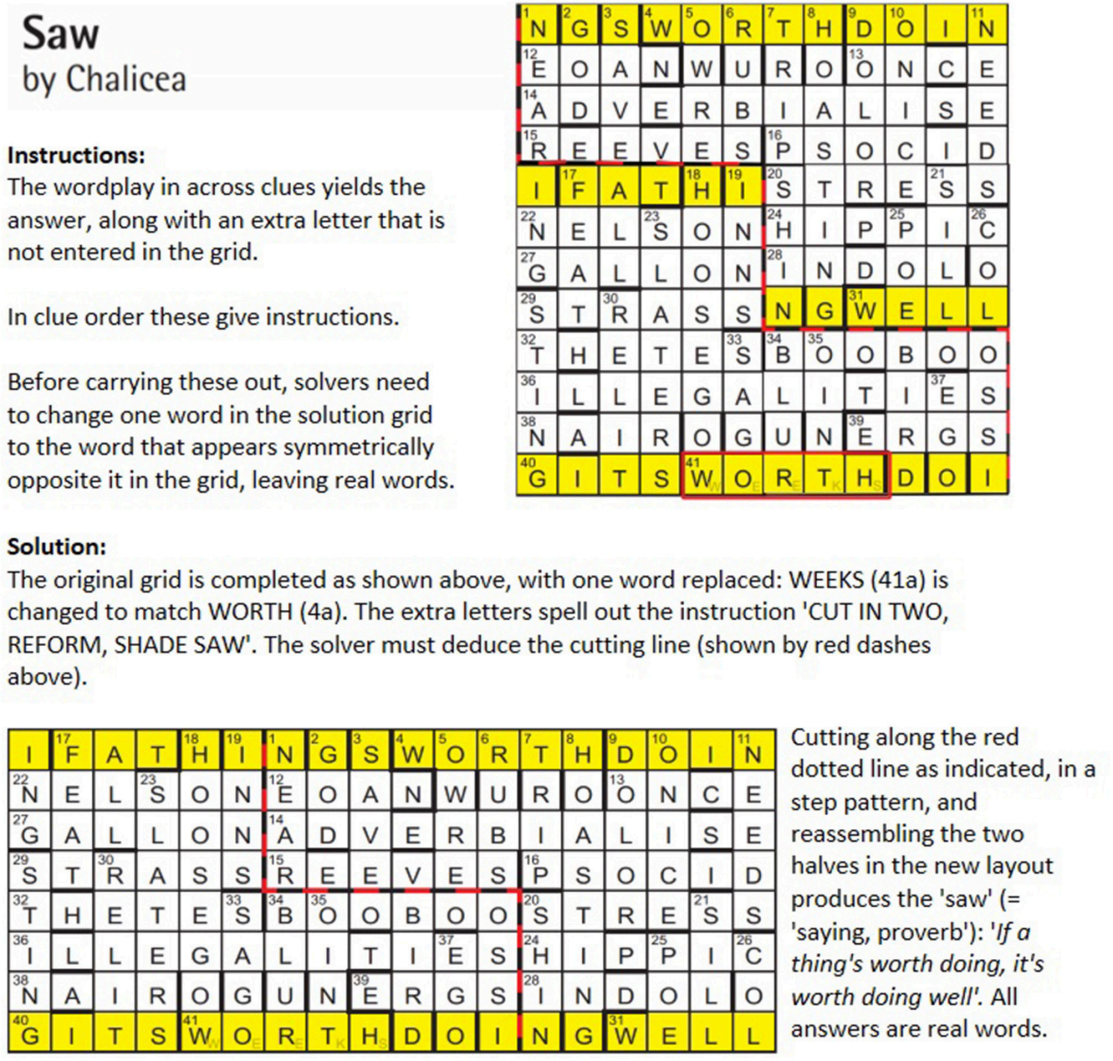
Magpie crossword issue 166.1 (Chalicea, 2016).

Further to this, some advanced cryptics require a type of restructuring in which the dimensions, layout or salient features of the grid itself are changed (see Figure 3). In these puzzles, there is a need to reconceptualize spatial assumptions involving placement and layout constraints, and to dismantle an existing array in favor of a new, radically different format. Cunningham highlights these two characteristics as strong features of classic spatially-oriented insight puzzles such as the nine-dot problem, the ten-coin triangle and the chain necklace puzzle (Cunningham et al., 2009 - Figure 1). Difficulty is also heightened in many of these classic puzzles by the need to identify and verify what the eventual solution would look like (MacGregor et al., 2001; Cunningham et al., 2009): this prevents steady progress toward a concrete and visualizable goal state (MacGregor et al., 2001), even if the eventual solution criteria and constraints are made clear.

So, for example, in Figure 3, the solver is made aware by means of a hidden message that the grid must be cut up and reassembled; but the purpose of this transformation, the eventual grid layout and even the cutting line must all be deduced. Additional difficulty is introduced by the elliptical reference to a “saw”; given the need to cut the grid and the zig-zag nature of the cut, the required interpretation of the term (“saw” = a maxim, saying) might not spring to mind. Without understanding this hint, the unspoken endgame (that of reconstructing a well-known phrase along the top and bottom line) cannot be interpreted correctly.

## Incidental support for cryptic crossword clues as a form of insight puzzle

The paper review set out above plausibly suggests that cryptic crosswords can function as insight problems, using a variety of techniques, such as misdirection and an ill-defined problem space, to increase the likelihood of an “Aha!” response. However, following the methodology set out in the “*Grounded Expertise Components Approach*” (GECA—Friedlander and Fine, 2016), the first step in the current research program was to secure empirically based corroboration for this *a priori* assumption.

Confirmation was therefore sought as part of an 84-item broad-based questionnaire, intended to characterize the cryptic crossword solving population across a wide number of dimensions. The full methodology for this research was set out in a previous publication (Friedlander and Fine, 2016). In total, 805 solvers across the full range of solving ability took part, although there was some attrition toward the end of the survey. Solvers were objectively assigned to research categories on the basis of benchmarked criteria, resulting in both a 2-way (Ordinary/Expert—O/E) and a 3-way (Ordinary/High ability/Super-Expert—O/H/S) categorization of participant expertise. For full details of the categorization rationale, see Friedlander and Fine, 2016.

One key hypothesis of the survey was that “cryptic crossword solving regularly generates ‘Aha!’ or insight moments, supporting the hypothesis that the cryptic clue is a type of insight problem through misdirection; and that this pleasurable experience is a salient driver of cryptic crossword participation” (Friedlander and Fine, 2016, p. 7). To this end, the survey included a number of questions pertinent to the current discussion: results are presented below. All chi-square analyses are bootstrapped and 95% confidence intervals are reported in square brackets.

## Evidence for the “penny-dropping moment” (PDM) and incubation effects

### PDM as a motivating experience

Participants were asked to rate 26 statements relating to their motivation for solving cryptic crosswords on a 5-point Likert scale (1 = “Completely Disagree”; 5 = “Completely Agree”). There were 786 responses (O: *n* = 388; H: *n* = 221; S: *n* = 177). Table 1A shows the five highest responses to these 26 statements (with abbreviated descriptions). As previously reported (Friedlander and Fine, 2016) all groups rated the “Aha!” moment (PDM) as a key motivational factor for solving cryptics; closely allied with this was the statement “Solving well-written clues gives me a buzz—it makes me smile or laugh out loud” which was ranked 4th in importance. The feeling of fulfillment—whether with the completed grid or with the “uniquely satisfying” cryptic crossword puzzle format—was also ranked highly (2nd and 5th most important). There were no statistically significant differences between the expertise groups for any of these statements. This suggests that—as for jokes—an important part of the crossword puzzle-solving experience lies in the pleasurable emotional reward bound up with the resolution of incongruity at the moment of insight. Studies of jokes and humor have found that laughter is associated with the release of endorphins which may be important in this context: the opiate effects of endorphins create a sense of wellbeing, pleasure and a sense of satisfaction (Dunbar et al., 2011). By contrast, extrinsic motivators, such as prizes, competitions, or public acclaim, were not important to participants across the board (Friedlander and Fine, 2016).

**Table 1:** Responses by expertise category to questions about “insight” properties of crossword clues.

	**O**	**H**	**S**	**All groups**
**(A) Top responses to “crossword motivation” question (mean scores, out of 5)**				
Number of responses to question	388	221	177	786
1. Enjoy “Penny-Drop Moment”	3.92	3.92	4.07	3.96
2. Cryptics are uniquely satisfying	3.89	4.05	3.91	3.94
3. Mental exercise to keep brain sharp	3.88	3.83	3.85	3.86
4. Makes me smile or laugh	3.79	3.80	3.64	3.76
5. Satisfaction of filled grid	3.46	3.61	3.36	3.48
**(B) % Participants agreeing with the following statements**				
Number of responses to question	395	223	178	796
“Setting the crossword aside for a while helps”^a^	95.7	95.5	91.1	94.6
-“sometimes, though not always”	12.4	15.7	16.8	14.3
-“always (answer is obvious on return)”	83.3	79.8	74.3	80.3
“I have solved clues when I'm doing something else”	77.5	79.8	84.8	79.8
“The Aha! feeling is most intense after a long struggle”				
- “Yes”	83.8	78.0	72.5	79.6
- “No difference one way or the other”	13.7	17.0	21.3	16.3

a *There were 797 responses to this question; S n = 179*.

### Incubation effect

In a separate series of questions intended to capture the solving preferences of participants, respondents were invited to rate statements on a 3-way Likert scale (“No/Never”-“Perhaps/Sometimes”-“Yes/Always”; together with a null response option “Don't know/Not applicable”). 796 responses were made (O: *n* = 395; H: *n* = 223; S: *n* = 178). Results are given in Table 1B: figures represent the summed percentage of “Sometimes” and “Always” responses unless otherwise indicated.

Nearly 95% of solvers (94.6%; O: 95.7%; H: 95.5%; S: 91.1%) confirmed that “incubation effects”—setting the crossword aside for a while, in order to resolve periods of impasse—were a feature of the solving process. Indeed, 80.3% of participants agreed with the full “Yes” option: “*Yes—the answer is often obvious when I return to the crossword”* with a further 14.3% agreeing that “*I sometimes find it helpful to take a break, but I often return to the thoughts I was having previously.”* S solvers were least likely to have taken advantage of incubation breaks; even so, differences in the distribution of incubation effect between groups failed to reach statistical significance ($\chi_{\left(4\right)}^{2}$ = 8.681, *p* = 0.070, Cramer's V = 0.074 [0.040, 0.135]).

Conversely, S participants were most likely (84.8%) to have found that solutions occurred to them at least occasionally when they were engaged in totally unrelated activities (e.g. shopping, driving, taking a bath). Overall 79.8% of participants agreed with this statement (O: 77.4%; H: 79.9%; S: 84.8%), but differences between the groups again failed to reach statistical significance ($\chi_{\left(4\right)}^{2}=5.393$, *p* = 0.249, Cramer's V = 0.058 [0.032, 0.115]).

### Impasse and the “Aha” moment

Most participants also agreed that their enjoyment of the PDM was enhanced if they had needed to struggle with a clue (79.6%; O: 83.8%; H: 78.0%; S: 72.5%) although some respondents claimed that the “Aha!” moment was unaffected by the effort expended (16.3%; O:13.7%; H: 17.0%; S: 21.3%). Very few participants claimed either that it decreased with effort expended (2.6%) or that they had never experienced a PDM (1.4%) when solving cryptics. Differences between groups approached, but did not achieve statistical significance ($\chi_{\left(6\right)}^{2}=11.796$, *p* = 0.067, Cramer's V = 0.086 [0.059, 0.153]) and inspection of standardized residuals indicated that this was driven by the higher number of S solvers in the “Makes no difference” group (*z* = 1.7).

## Differences in solving approach between cryptic crossword expertise groups

Participants were also asked about their approach to solving cryptics in order to explore potential differences between the expertise groups; Table 2 highlights a number of key findings.

**Table 2:** Differences in approach to solving cryptics.

	**O**	**H**	**S**	**All groups**
Number of responses	395	223	179	797
**(% Participants agreeing with the following statements)**				
**(A) Do you notice the surface reading or the codes of a clue first?**				
Surface first	33.2	25.6	25.7	29.4
Bit of both: surface and codes	50.4	42.6	38.0	45.4
Read as code, not for meaning	16.5^***^	31.8^*^	36.3^**^	25.2
**(B) What do you look for in an Advanced Cryptic crossword?**^a^				
I don't do Advanced Cryptics	n/a	12.1^*^	2.8^*^	8.0
Great clues	n/a	35.9^*^	16.8^**^	27.4
Good balance of clues and endgame	n/a	38.6	50.3	43.8
Tricky and satisfying Endgame	n/a	13.5^*^	30.2^**^	20.9
**(C) Are you disappointed if you solve a crossword rapidly?**^b^				
No: I enjoy rapid solving	9.9	14.3	16.9	12.7
Don't mind either way	41.8	33.6	41.0	39.3
Yes: I like to wrestle with the clues	48.4	52.0	42.1	48.0
**(D) I Would change my crossword if the challenge got too easy (“Yes”)**	70.1	71.7	66.3	69.7

(*/**/*** *indicates significance at the 0.05/0.01/0.001 level)*.

a *Ordinary solvers, by definition, do not solve Advanced Cryptic crosswords. %s relate to 402 participants (H = 223; S = 179)*.

b *There were 796 responses to this question; S n = 178*.

### Suppression of the misleading surface reading

Survey participants were asked to indicate whether they noticed the surface reading of a clue first, or read it purely as code. Two response options (“I always read the surface meaning first,” “I tend to read the surface first”) favored the surface reading; two options indicated that deliberate attempts were made to exclude “reading for sense” (“I try to exclude the misleading context,” “I always read as code: the surface meaning could be gobbledygook”); and there was one mid-way option (“Bit of both; not sure which predominates”). There were 797 responses (O: *n* = 395; H: *n* = 223; S: *n* = 179); summarized details (Surface/ Bit of Both /Code) are given in Table 2A.

Most solvers (45.4%; O: 50.4%; H: 42.6%; S: 38.0%) selected the mid-way point, though this decreased with expertise: S solvers were most likely to suppress “reading for sense” in favor of “reading for code” (36.3%); the opposite was true for O solvers, who tended to read much more for sense (33.2%). Differences between the groups were significant ($\chi_{\left(4\right)}^{2}=33.21$, *p* < 0.001, Cramer's V = 0.144 [0.105, 0.199]) and inspection of standardized residuals indicated that this was driven by higher levels of H (31.8%, z = 2.0, *p* < 0.05) and S (36.3%, z = 3.0, *p* < 0.01) solvers who suppressed the surface reading; and lower levels of O solvers who did this (16.5%, z = −3.5, *p* < 0.001).

### Personal preferences leading to greater enjoyment of advanced cryptic crosswords

Solvers were asked to identify whether they solved Advanced Cryptic crosswords, and, if so, whether the quality of the clueing or the tricky endgame (or a bit of both) was their primary source of enjoyment (Table 2B). A small proportion of both expert groups chose not to solve Advanced Cryptic crosswords, although this was higher for H solvers than for S (“I don't do Advanced Cryptics”: 8.0%; H 12.1%; S 2.8%). O solvers, by definition, do not solve this type of crossword (Friedlander and Fine, 2016, p. 8) and were omitted from this analysis. Where a preference was indicated, for H solvers the quality of the clueing was paramount (27.4%; H 35.9%; S 16.8%) whereas, for a larger number of S solvers, the lateral-thinking endgame was the most important attraction (20.9%; H 13.5%; S 30.2%). Differences between the groups were significant ($\chi_{\left(3\right)}^{2}=40.47$, *p* < 0.001, Cramer's V = 0.317 [0.226, 0.407]) and inspection of standardized residuals indicated that this was driven by higher levels of H (12.1%, z = 2.2, *p* < 0.05) and lower levels of S (2.8%, z = −2.5, *p* < 0.05) who failed to tackle Advanced Cryptics; higher levels of H (35.9%, z = 2.4, *p* < 0.05) and lower levels of S (16.8%, z = −2.7, *p* < 0.01) whose main target for enjoyment was the smooth clueing; and higher levels of S (30.2%, z = 2.7, *p* < 0.01) and lower levels of H (13.5%, z = −2.4, *p* < 0.05) whose primary focus was the endgame.

### Speed-solving and challenge

Solvers were also asked whether they would be disappointed if they solved a crossword rapidly (Table 2C). Although chi-square showed a significant association overall ($\chi_{\left(4\right)}^{2}=9.99$, *p* = 0.041, Cramer's V = 0.079 [0.050, 0.139]), inspection of the standardized residuals revealed no stand-out elements. As expected, S solvers (among whom were a number of competition-focused “Speed Solvers”—see Friedlander and Fine, 2009) would be least troubled by a rapid solve (“No: I enjoy speed-solving”: 12.7%; O 9.9%, z = −1.6; H 14.3%, z = 0.7; S 16.9%, z = 1.6), but, even for this group, numbers were low, and standardized residuals were non-significant. Nearly half the solvers indicated that they would be disappointed without a good challenge to wrestle with, and although there was some variation across the expertise groups (48.0%; O 48.4%, z = 0.1; H 52.0%, z = 0.9; S 42.1%, z = −1.1) inspection of the standardized residuals were once again non-significant.

Indeed, when asked whether they might switch newspapers if the crossword challenge became routinely easy (Table 2D), nearly 70% of solvers indicated that they would consider this (69.7%; O 70.1%; H 71.7%; S 66.3%), with differences between the groups being statistically non-significant.

## Potential contribution of cryptic crosswords to insight research

The above review suggests that the cryptic crossword domain could prove a useful addition to the repository of insight problem paradigms. That they are capable of triggering insight on a regular basis is quite clear: survey results reported above indicate that cryptic crossword solvers were primarily motivated to solve cryptics because of the “Aha!” or “Penny-Drop” moment, and also reported that the “laugh-out-loud” moment at the point of solving the clues was highly enjoyable. Furthermore, the detailed review of cryptic clues set out above demonstrates that they use a broad variety of insight-triggering mechanisms shared in common with a wide range of other insight problem formats. A single cryptic crossword puzzle thus presents a unique compendium of heterogeneous challenges which sets it apart from all other methodologies currently available; and this should facilitate the comparison of outcomes between device types within the crossword itself, as well as with other insight puzzle challenges external to the crossword.

One small caveat is that cryptic crosswords are primarily restricted to a number of English language speaking countries, although a few cryptic type puzzles do exist in Dutch and German. This may reduce the flexibility of cryptic crosswords as an insight puzzle paradigm. Straight-definition crosswords are, of course, available in all languages, but lack the cryptic elements described in detail in this paper which set this puzzle form apart and trigger the insight moment.

Cryptic crossword clues thus reliably trigger insight experiences, but (as for all insight puzzles) this is not exclusively the case. In cryptic crossword trials filmed for transcription using Verbal Protocol Analysis (VPA), casual inspection of the recordings suggests that not every clue produces as many PDMs; and not every solver follows the same path to solution. Systematic analysis of the video recordings (on which see further Friedlander and Fine, 2016) will allow us to take full advantage of the think-aloud protocol to capture a wide range of strategically important factors such as intuitive vs. analytical approaches to clue solution; the length of time spent in impasse on each clue before moving onto another; the frequency of return to an obstinately resistant item; perseveration with an incorrect solution pathway; the antecedents of “Aha!” solution moments; the use of cross-checking letters as opportunistic solution prompts; the suppression of the surface meaning on initial reading; the certainty of correctness (without double-checking) on solution; and the use of jottings such as candidate anagram letters (see Box 5 above) to facilitate solution (on the use of VPA in the GECA methodological approach, see further Friedlander and Fine, 2016). These aspects are all highly relevant to the discussion of insight problem solving across a wide range of problem domains.

As a precursor to the analysis, the clues used in the crossword trials will be individually analyzed to identify salient features, such as the mechanisms employed, the level, and number of the constraints preventing solution, and the predicted difficulty which flows from this (following e.g., Knoblich et al., 1999; Cunningham et al., 2009; MacGregor and Cunningham, 2009). It is very possible that the clues vary in difficulty on a principled basis, and if so, this might lead to a better understanding of what makes a cryptic crossword clue enjoyable, and more likely to trigger insight, to lead to impasse, or to invoke “Immediate Insight” solutions. Given the cross-over between cryptic crossword clue types and other insight puzzles, this should shed helpful light on insight mechanisms in other areas, too.

Logistically, cryptic crosswords also offer a number of advantages over other puzzle types. In the first place, there is no lack of material: cryptic crosswords appear daily in all of the British newspapers, and widely across the world in countries with historically strong connections to Britain (e.g., Canada, Ireland, Australia, New Zealand, India, and Malta: Friedlander and Fine, 2016). It is thus entirely possible to commission a professionally composed, high-quality puzzle specifically for a research study thus guaranteeing that all participants will be naïve to the challenge. Clue solution rates are high, too: in trials involving 28 solvers (both expert and non-expert) tackling a commissioned 27-clue crossword of medium difficulty, 682 of the 756 clues (90.2%) were solved correctly within the 45 min time limit (Fine and Friedlander, in preparation). Solving times for those who finished the entire puzzle (*n* = 19) could be very rapid indeed (range solving times: 10m47s−40m30s; mean solving time for finishers 23m:43s, median 22m15s) resulting in solutions occurring, on average, approximately once a minute (Fine and Friedlander, in preparation).

Fast solvers in this trial were all highly expert in the field (Fine and Friedlander, in preparation), and the survey results set out above also indicate that experts may approach the solving of cryptic clues in subtly different ways to less expert solvers of equivalent experience. What could be seen as a disadvantage for this methodology (that cryptic crossword solving is a niche activity requiring inside knowledge of and experience with the clue mechanisms) thus becomes a compelling strength: there is much that might be gained from studying expert insight puzzle solvers at work, and this is currently impossible in other insight domains (such as RAT puzzles or matchstick math) which, by necessity, always use naïve populations.

Lamenting the lack of expertise studies in the insight area, Batchelder and Alexander (2012) even suggested artificially training groups of individuals to produce “expert” solvers of such problems, commenting that experts “might have the capacity to rapidly shift their search spaces until the type of space that contains the solution occurs to them” (Batchelder and Alexander, 2012, p. 88). However, this proposal overlooks the potential role of individual differences: MacGregor and Cunningham argue that there may be reliable variations in the ability of individual subjects to solve insight problems (2008; see also DeYoung et al., 2008; Ovington et al., 2016) which may undermine the ecological validity of training “experts” from a randomly selected sample of individuals. Within the crossword field we found naturally-occurring expertise groupings—all with equivalent levels of experience over many decades in the field, but with quite different expertise outcomes (Friedlander and Fine, 2016)—and this presents a unique opportunity for exploration.

The cryptic crossword survey data set out in Tables 1, 2 above hints at some interesting differences between the various expertise groups and their approach to solving this form of puzzle. Most intriguing of all is the possibility that experts have an enhanced capacity to resist the red-herring set for them, by electively divorcing the reading of the clue from its surface meaning (“the surface meaning could be gobbledygook”), and thus shielding the mind from the deliberate misdirection. Whether expert solvers therefore experience the full phenomenological experience of the “Aha!” moment upon solution of the clue is thus an interesting angle for further investigation: experts claim to be equally motivated by the promise of the “Aha!” moment (Table 1), yet, paradoxically, appear to suppress that very need for Representational Change which might have been considered fundamental to the insight experience. Experts also solve more rapidly, with speed prowess being a primary focus for some (Friedlander and Fine, 2009), and this affords an opportunity to explore rapid “pop-out” solutions and the relevance of “Immediate Insight” to the exploration of the “Aha!” moment.

It is also notable that significantly more Super-Experts engage in Advanced Cryptic puzzles than High Expert solvers, and that their primary focus in doing so is significantly more often linked, not with the appreciation of the smooth misdirection of the clueing itself, but with the complexity, novelty and lateral thinking challenge of the Advanced Cryptic endgame, which is more akin to the “classic” insight puzzle format in its use of thematic or spatial features. This again affords opportunities to examine the multi-dimensional nature of the demands posed by different insight problem types, as described in the body of this article, and the interplay with individual differences shown by problem solvers, in terms of their thinking and personality styles.

## Conclusion

In sum, this preliminary review suggests that cryptic crossword puzzles may be a promising source of insight problems offering a number of potential advantages over some of the puzzles and riddles previously used: for example, they are readily obtainable in potentially unlimited supply, solvable within acceptable time limits and suited to the simultaneous exploration of a variety of puzzle types and their potentially distinct solving mechanisms. Uniquely among existing paradigms, they also afford us the opportunity to study insight-solving expertise in action and to identify the characteristics and methodological approaches of those with a particular propensity to solve these puzzles effectively. There is therefore much to explore, and the discussion above suggests a number of particularly interesting avenues which we are currently pursuing. We believe that this new paradigm may prove to be a useful source of theoretically and empirically grounded, heterogeneous insight challenges; and that it is well-placed to shed a unique light on the workings of this elusive and intriguing aspect of human cognition.

## Ethics statement

This study was carried out in accordance with the recommendations of the British Psychological Society. All subjects gave written informed consent in accordance with the Declaration of Helsinki. The protocol was approved by the School of Science and Medicine Ethics Committee, University of Buckingham.

## Author contributions

KF drafted the article and KF and PF reviewed and finalized it. KF designed the survey and analyzed data via an Access database. KF and PF reviewed data and agreed coding treatments.

### Conflict of interest statement

The authors declare that the research was conducted in the absence of any commercial or financial relationships that could be construed as a potential conflict of interest.

## Footnotes

^1^http://www.piemag.com/about/

^2^http://www.listenercrossword.com/List_Awards.html
